# An open-source test stand for backlash measurement in low-cost UART servo motors

**DOI:** 10.1016/j.ohx.2026.e00767

**Published:** 2026-03-28

**Authors:** Boris Kotov

**Affiliations:** Robonine, United States of America

**Keywords:** Backlash measurement, Servo actuators, Open-source hardware, Automated test stand, UART serial bus, Low-cost instrumentation

## Abstract

Backlash in compact servo actuators is a common source of positioning error in low-cost robotic and mechatronic systems. Measuring this backlash reliably is difficult because most servos include only a single output-shaft encoder, and conventional tools such as dial indicators can introduce enough probing force to distort the measurement.

We present an open-source test stand that applies small, repeatable loads to a servo lever and measures the resulting displacement under both loaded and unloaded conditions. The stand uses low-cost components, 3D-printed fixtures, interchangeable levers, and a soft elastic coupling to apply controlled forces in opposite directions. Accompanying software coordinates the test sequence, records telemetry, and analyzes backlash using a consistent, repeatable methodology.

We demonstrate how the platform can characterize single-servo and coupled-servo configurations, enabling direct comparison of mechanical performance and aiding actuator selection in design work. All CAD files, control software, and analysis tools are openly provided to support replication and further development.

## Graphical abstract

**Figure:**
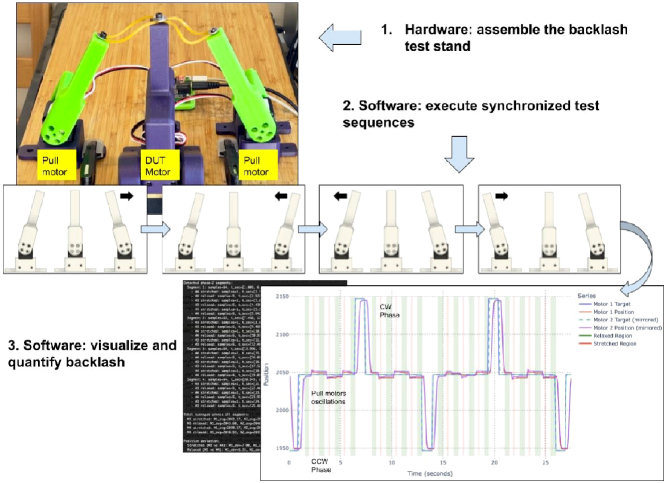

## Specifications table

**Table:** 

Hardware name	Servo Backlash Measurement Test Stand
Subject area	•Engineering and material science • Educational tools and open source alternatives to existing infrastructure

Hardware type	•Mechanical engineering and materials science

Closest commercial analog	Manual dial indicator or dial test indicator backlash measurement setup ($50–$200, manual operation, probe contact force). No commercially available low-cost automated system exists for backlash measurement in assembled UART serial bus servo actuators.

Open source license	CC-BY-4.0 (hardware and paper)
GNU GPL-3.0 (software)	

Cost of hardware	Approximately $100 USD (complete test stand)

Source file repository	DOI: https://doi.org/10.5281/zenodo.19261714 Zenodo: https://zenodo.org/records/19261714 (GitHub mirror: https://github.com/roboninecom/Measuring-Backlash-in-Popular-UART-Servos)

## 1 Hardware in context

Low-cost servo motors have become a common choice in DIY robotics, hobby mechatronics, and education. UART-controlled servos — such as those from *Feetech* and *Waveshare* — are popular because they offer digital configuration, compact size, and affordable pricing [1], [2]. Their simplicity makes them easy to integrate into small research setups and classroom projects.

Despite these advantages, such servos have important limitations. Most notably, their geartrains introduce mechanical backlash, creating a region where changes in the commanded position do not immediately move the output shaft. Even a small amount of backlash can reduce positioning accuracy, degrade repeatability, or lead to oscillatory behavior. In addition, these servos typically include only a single encoder on the output shaft (e.g., 12-bit output encoders are commonly specified for this class), so internal gear motion cannot be observed directly and must instead be inferred from output behavior [1], [2], [3].

Backlash in small servos is often measured using a dial indicator (or a lever-type dial test indicator) at the lever tip. While convenient, this method introduces several sources of error: the probe applies a small but non-negligible force, which can shift the output shaft and mask the true backlash. Manufacturer specifications for common indicators report measuring forces on the order of 0.2–0.4 N for dial test indicators and up to 1.8 N for plunger-type dial indicators, making probe-induced torque nontrivial for lightweight actuators [4], [5]. Friction in the indicator mechanism, inconsistent contact pressure, and difficulty keeping the lever aligned with the measurement axis add further uncertainty.

More advanced backlash identification techniques have also been reported, including inferential and drive-side methods (e.g., momentum-transfer approaches, current-signal inference, and model-based estimation; [6], [7], [8]. While these approaches can be effective in their intended contexts, they often increase implementation complexity (instrumentation, parameter identification, signal processing, or experimental setup) and are not designed as low-cost, easily replicable hardware platforms for routine characterization of assembled compact UART serial bus servos.

To address this gap, we present an open-source test stand that prioritizes (i) low cost and replicability, (ii) automated, repeatable bidirectional loading, and (iii) eliminating probe contact force by using the servo’s own output-shaft encoder as the measurement device. The stand applies small, repeatable loads to a servo lever and measures the resulting displacement under both loaded and unloaded conditions using a consistent, scripted procedure.

Table 1 summarizes representative approaches to backlash characterization in terms of cost, automation, and measurement interaction. The listed methods are not directly equivalent in what they measure (gear components vs. assembled actuators), but provide context for why a low-cost automated actuator-oriented platform is useful.

**Table 1:** Comparison of representative backlash measurement approaches.

Method	Cost	Resolution	Probe Force	Automation
*Industrial gear metrology (component-focused)*				
Gear metrology systems (e.g., Gleason GMS series) [9]	$20,000+	<0.01 °	None	Full

*Gear rolling testers (component-focused)*				
Double-flank gear rolling testers (manual/motorized)^a^[10]	$750–16,500+	1 µm^b^	None	None–Full

*Dial-indicator-based methods (assembled or fixture-based)*				
Dial test indicator (lever-type; e.g., Mitutoyo 513-series) [4]	$50–150	0.01 mm	0.2–0.4 N	None
Dial indicator (plunger-type; e.g., Mitutoyo 3414S) [5]	$80–200	0.01 mm	1.8 N max	None

**This work (assembled actuator-focused)**	$80–100	0.09°^c^	None	Full

a Representative commercial category; specific offerings vary by manufacturer and configuration.

b Linear resolution; angular resolution depends on gear geometry and setup.

c Limited by DUT encoder resolution (12-bit, 4096 counts/rev).

## 2 Hardware description

The test stand is designed to be simple, modular, and easy to reproduce. It can be configured to test either a single servo motor or two mechanically coupled servos. The mechanical system consists of a base plate, servo holders, 100 mm bracket levers, puller motors, the motor or motors under test, and elastic bands that provide a soft loading mechanism. The software subsystem includes test-control software for automated sequencing and telemetry logging, and analysis scripts for backlash calculation and visualization.

**Fig. 1:**
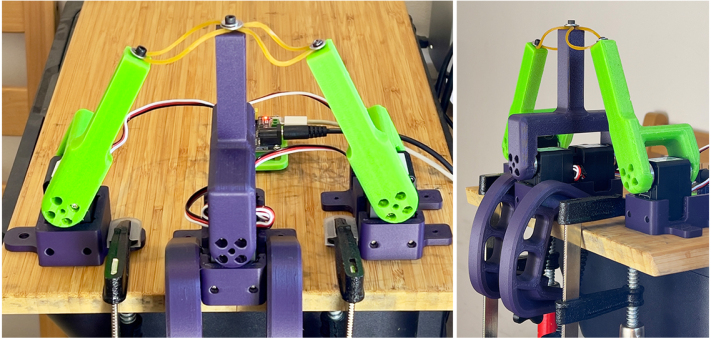
Test stand setup for testing two servos coupled via common bracket lever.

An overview of the complete mechanical setup is shown in Fig. 1.

This open-source test stand enables researchers and practitioners to:

• Measure backlash in assembled **UART servos** under controlled clockwise/counter-clockwise loading, without dial-indicator probe contact.
• Compare **UART servo** models and configurations (single vs. coupled servos; with/without pretension) using the same automated test sequence.
• Evaluate mechanical anti-backlash approaches (e.g., dual-motor biasing) by quantifying the reduction in the effective dead zone.
• Record repeatable telemetry logs for statistical analysis (cycle-to-cycle variation, drift, and sensitivity to load level).
• Reuse the fixtures and scripts for other compact bus servos by adjusting mounts, lever geometry, and communication settings.

In terms of cost and ease of use, the test stand is designed to be assembled from commodity components and 3D-printed fixtures, and operated with widely available software tooling (Node.js scripts for test control and Python scripts for analysis). The total cost of the base system is approximately $55-60 USD (excluding the DUT servo(s)), enabling use in small laboratories, teaching settings, and rapid prototyping workflows.

The platform supports both single-servo DUT testing (baseline backlash characterization) and dual-servo DUT testing (evaluation of coupled/pretensioned configurations intended to reduce backlash). In the dual-servo configuration, two servos are mechanically coupled and biased with opposing position offsets so that each gear train remains seated on opposite flanks, reducing the effective dead zone.

The test stand operates by applying alternating tensile forces to a lever arm attached to the DUT. Two puller motors, positioned on opposite sides, tension elastic bands that connect to the DUT lever. By alternately activating each puller, the system applies bidirectional torque to the DUT, causing the gear train to shift between opposite flanks. The resulting position deviation, measured by the DUT’s encoder, quantifies the backlash.

The elastic bands serve as compliant coupling elements that:

• Prevent impact loading that could damage gears
• Allow controlled force application through puller motor position
• Decouple the measurement from puller motor positioning errors

The design files and software are structured to facilitate adaptation. Mechanically, the DUT holder and lever arms can be reparameterized for different servo footprints and horn standards. Electrically, the serial bus and control scripts can be adapted to alternative UART bus servos and baud rates. Methodologically, the same loading-and-release principle can be extended by adding calibrated load sensing (e.g., an inline spring scale or load cell) to quantify backlash as a function of applied torque, or by scripting automated sweeps over multiple load levels and positions.

## 3 Design files summary

The main mechanical, control, and analysis files used in this work are summarized in Table 2.

**Table 2:** Project file structure overview.

File name	File type	License	Location
*Test Stand Mechanical Components*			

servo_holder_single.step	CAD (STEP)	CC-BY-4.0	/cad/
servo_holder_single.stl	3D Print (STL)	CC-BY-4.0	/cad/
test_lever_100mm_single.step	CAD (STEP)	CC-BY-4.0	/cad/
test_lever_100mm_single.stl	3D Print (STL)	CC-BY-4.0	/cad/
servo_holder_dual.step	CAD (STEP)	CC-BY-4.0	/cad/
servo_holder_dual.stl	3D Print (STL)	CC-BY-4.0	/cad/
test_lever_100mm_dual.step	CAD (STEP)	CC-BY-4.0	/cad/
test_lever_100mm_dual.stl	3D Print (STL)	CC-BY-4.0	/cad/

*Software – Test Control and Data Acquisition*			

app.js	Node.js application	GNU GPL-3.0	/software/backlash_test/
config.js	Node.js module	GNU GPL-3.0	/software/backlash_test/
sweepConfig.js	Node.js module	GNU GPL-3.0	/software/backlash_test/
TelemetryLogger.js	Node.js module	GNU GPL-3.0	/software/backlash_test/
package.json	Build configuration	GNU GPL-3.0	/software/backlash_test/

*Software – Data Analysis and Visualization*			

config/	Example configuration files (directory)	GNU GPL-3.0	/software/logs_analysis/
config_utils.py	Python script	GNU GPL-3.0	/software/logs_analysis/
log_calc.py	Python script	GNU GPL-3.0	/software/logs_analysis/
log_viz.py	Python script	GNU GPL-3.0	/software/logs_analysis/

*Project Data and Documentation*			

logs/	Telemetry CSV output (directory)	CC-BY-4.0	/logs/
README.md	Project documentation	CC-BY-4.0	/

## 4 Bill of materials

**Cost summary (USD):**

**Table 3:** Bill of materials summary for the backlash measurement test stand (base system and optional DUT configurations).

Designator	Component	Qty	Unit cost (USD)	Source (link)	Material type
*Actuators*					
M1–M2	Feetech STS3215 servo (puller motors)	2	18.00	AliExpress^a^	Other
M3	Feetech STS3215 servo (single-servo DUT option)	1	18.00	AliExpress^a^	Other
M3–M4	Feetech STS3215 servo (dual-servo DUT option)	2	18.00	AliExpress^a^	Other

*3D-printed fixtures*					
P1–P2	servo_holder_single.stl	2	2.00	Self-printed	Polymer
P3–P4	test_lever_100mm_single.stl	2	2.00	Self-printed	Polymer
P5	servo_holder_single.stl (single DUT option)	1	2.00	Self-printed	Polymer
P6	test_lever_100mm_single.stl (single DUT option)	1	2.00	Self-printed	Polymer
P7	servo_holder_dual.stl (dual DUT option),	1	3.00	Self-printed	Polymer
P8	test_lever_100mm_dual.stl (dual DUT option)	1	3.00	Self-printed	Polymer

*Elastic loading*					
E1–E2	Elastic bands, rubber, ∼100 mm	2	0.50	Office supply^b^	Polymer

*Electronics and wiring*					
U1	USB–TTL adapter, e.g. Feetech FE-URT-1	1	5.00	AliExpress^c^	Semiconductor
PS1	12 V DC power supply, ≥2 A, barrel connector	1	8.00	Amazon^d^	Other

*Fasteners (dual DUT option)*					
F1–F3	M3 × 10 screw, stainless steel	3	0.10	Hardware store^e^	Metal
F4–F11	M4 × 16 screw, stainless steel	8	0.12	Hardware store^e^	Metal
F12–F19	M4 hex nut, stainless steel	8	0.05	Hardware store^e^	Metal

**Notes:** (i) “Self-printed” items refer to parts whose STL/STEP files are provided in the design files section; the listed costs reflect estimated material cost per print.

a AliExpress listing/search for “Feetech STS3215 serial bus servo” (example): https://www.aliexpress.com/wholesale?SearchText=Feetech+STS3215+serial+bus+servo.

b Example: rubber band assortment (100 mm class) available from general office suppliers (search): https://www.amazon.com/s?k=rubber+bands+100mm.

c AliExpress listing/search for “USB TTL FT232RL 3.3V” (example): https://www.aliexpress.com/wholesale?SearchText=Feetech FE-URT-1 FE-URT-1.

d Example: “12 V 2 A power supply barrel” (search): https://www.amazon.com/s?k=12v+2a+power+supply+barrel.

e Example: “M3 stainless screws and nuts” (search): https://www.mcmaster.com/metric-screws/m3/.

• Base system (pullers + fixtures + electronics): **$58.00**
• With single-servo DUT option: **$80.00**
• With dual-servo DUT option: **$101.66**

## 5 Build instructions

Throughout this section, component designators (e.g., M1, P3, U1) refer to the bill of materials in Table 3.

### 5.1 Hardware overview

#### 5.1.1 Tested motor(s)

The center of the stand holds either one tested servo or a pair of coupled servos. The tested servo(s) are mounted in the corresponding single- or dual-motor holder and fitted with a 100 mm lever. This lever serves as the force-application point.

#### 5.1.2 Puller motors

Two puller motors apply controlled external forces to the tested motor or motors. These motors may be Feetech or Waveshare UART servos, and they are mounted in simple 3D-printed holders on the base plate. Each puller motor is fitted with its own 100 mm lever, aligned so that force can be applied cleanly to the lever of the tested servo.

The puller motors do not participate in measurement. Their primary role is to generate repeatable clockwise and counter-clockwise loading during the test sequence. However, their position data is still logged and later used by the analysis software to determine the active phase of the test (preload, loaded, or unloaded) and to correctly classify samples for backlash estimation.

#### 5.1.3 Elastic bands for soft coupling

Elastic bands provide a soft and backlash-free connection between the puller motors and the tested motor lever. In the neutral position, the bands are fully relaxed. The puller motors rotate to a predefined angle to tension the bands and apply the desired force during the test.

Using elastic bands as the coupling element prevents additional mechanical backlash from being introduced into the system and smooths transitions when the load direction changes. The applied force is calibrated before testing by measuring the pull at the lever tip, allowing different force levels to be used depending on whether a single motor or a coupled pair is being evaluated.

### 5.2 Prerequisites

The test stand is designed so that all major assembly steps — mounting servos in holders, attaching levers, and wiring — use only the screws and cables provided with the servos. No special fasteners are required except for those used to attach holders to the base plate and to secure elastic bands at the lever tips.

Basic assembly requires only standard hand tools, such as a small Phillips screwdriver, hex keys for M3 hardware, and optionally clamps for fixing holders to the base plate. A hanging scale is recommended for calibrating the elastic-band force but is not needed for basic mechanical assembly.

#### 5.2.1 Motor fixtures

Most UART servos used in this test stand (e.g., Feetech and Waveshare models) are supplied with the basic hardware required for mechanical assembly. These typically include:

• 25T output horns (disks) for attaching levers
• Thread-forming screws for securing the servo body within mounting holders
• M3 machine screws for fastening 25T horns and levers to the output shaft
• Daisy-chain cables for connecting multiple servos to a shared UART bus

Before further assembly, 25T output disks (horns) must be attached to each puller servo.

#### 5.2.2 3D printed parts

The stand uses several 3D-printed parts, including servo holders and 100 mm levers (see the design files in Table 2). For assembly, these correspond to holders P1–P2 and levers P3–P4 (pullers), and holder/lever P5–P6 (single-servo DUT) or P7–P8 (dual-servo DUT), as listed in Table 3. Note that the puller levers (P3–P4) and the single-DUT lever (P6) are the same printed part (test_lever_100mm_single.stl); likewise P1–P2 and P5 all use servo_holder_single.stl.

Parts can be printed on a standard FDM printer using common materials such as PLA or PETG; moderate infill (e.g., 30%–40%) is sufficient for typical loads.

Material choice (PLA vs. PETG) does not affect the measurement principle because load is applied via elastic bands and backlash is computed from the DUT encoder shift; printed parts serve only as fixtures requiring geometric repeatability and sufficient stiffness at the tested loads.

#### 5.2.3 Base plate

The base plate serves as the foundation of the stand. It is a flat, rigid surface without any predefined mounting patterns. Components are attached using clamps or fasteners, depending on the material and the required rigidity. This approach keeps the design flexible and allows users to adapt the layout to different test configurations. Materials such as acrylic, plywood, aluminum, or 3D-printed panels can all be used successfully.

#### 5.2.4 Hanging scale

Any small scale with a hook (e.g., a spring scale or a compact digital hanging scale) can be used to calibrate the tensile force applied to the DUT lever by the elastic bands. During calibration, the hook is attached to the elastic band at the same point where it connects to the lever, and the band is stretched *parallel to the ground* while reading the force on the scale. The band extension (or, equivalently, the lever-to-lever distance between the puller and DUT attachment points) is then adjusted until the target force is reached. Recording this extension distance provides a simple, repeatable reference that can be reproduced when positioning the puller mounts, reducing setup-to-setup variability after disassembly and reassembly.

#### 5.2.5 Servo ID assignment

Each servo must be assigned a unique ID before installation. This is done using the Feetech configuration utility FD 1.9.8.3 [11].

1. Connect a single servo to the USB–TTL UART board using one of its UART ports.
2. Connect the power supply to the UART board, then connect the board to the PC via USB.
3. Open FD 1.9.8.3, select the correct COM port and baud rate (typically 1,000,000 bps for Feetech/Waveshare UART servos) and click **Connect**.
4. Click **Scan** to detect the connected servo.
5. Open the Programming tab, assign a new servo ID (register 5), and **Save** the settings.

**Fig. 2:**
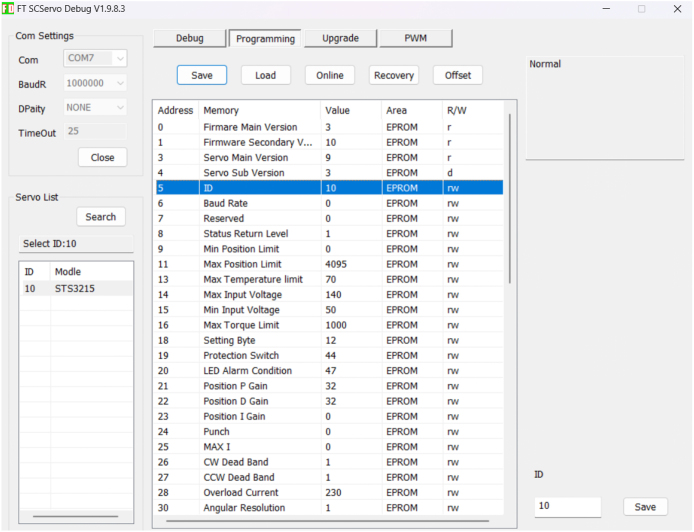
Servo ID assignment in the Feetech FD configuration utility.

Repeat this process for each servo (puller servos and tested servo(s)) to ensure all IDs are unique before assembling the test stand. Fig. 2 shows the relevant screen in the FD utility used to scan the bus and write a new ID to the servo.

### 5.3 Hardware assembly

#### 5.3.1 Install the puller servos

1. Place the puller servos (M1–M2) into the single-servo holders (P1–P2).
2. Secure each servo using the thread-forming screws supplied with the servos.
3. Attach a 100 mm lever (P3–P4) to each puller servo using the servo-supplied M3 × 5 screws. Ensure the lever is firmly seated on the 25T horn.

**Fig. 3:**
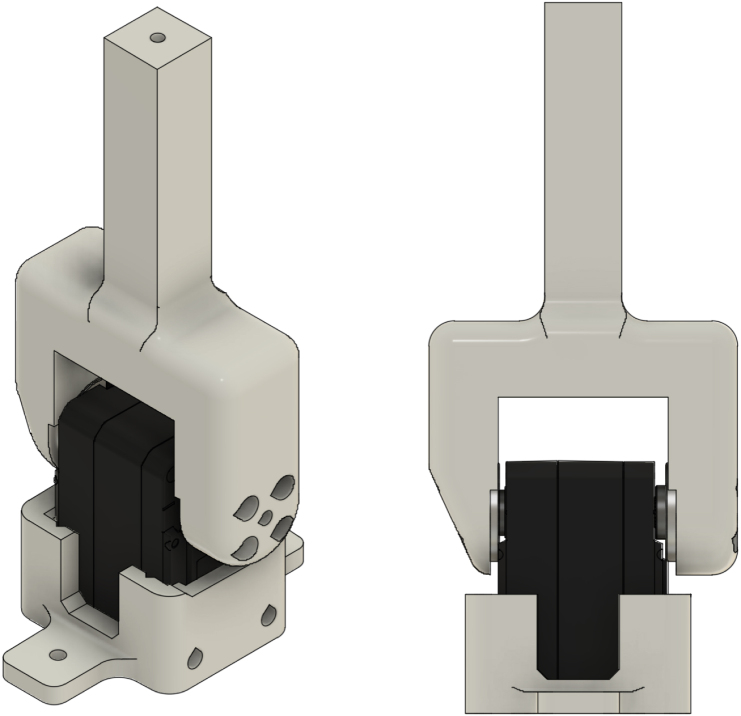
A completed puller-servo assembly (holder + servo + 100 mm lever).

Fig. 3 shows a completed puller-servo assembly used on both sides of the stand to apply bidirectional tensile loading via elastic bands.

#### 5.3.2 Install the tested servo(s)

The stand supports either a single tested servo or two coupled servos.

1. For a single-servo DUT, place the tested servo (M3) into the single-servo holder (P5). For a dual-servo DUT, place both servos (M3–M4) into the dual holder (P7).
2. Secure the servo(s) using the provided thread-forming screws.
3. Attach the appropriate 100 mm lever: P6 for a single-servo DUT, or P8 for a dual-servo DUT.
4. Tighten the lever screws using the servo-supplied M3 × 5 screws.

**Fig. 4:**
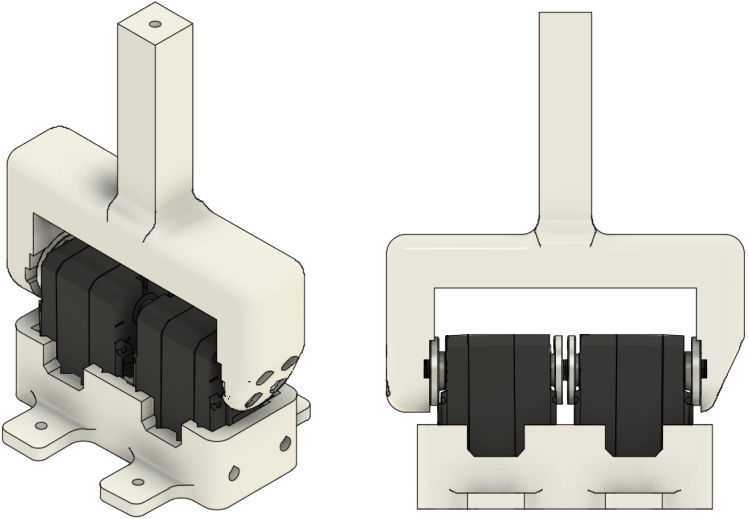
Dual-servo DUT configuration: two UART servos mounted in the dual holder and rigidly coupled via the dual test lever.

A dual-servo lever configuration is illustrated in Fig. 4. This arrangement enables evaluation of coupled/pretensioned configurations intended to reduce effective backlash.

#### 5.3.3 Attach elastic bands

1. Use an M3 × 10 screw (or any similar small screw/bolt available to you) as an anchor point at the lever tip.
2. Hook the elastic bands (E1–E2) onto the anchor and connect them between the puller-motor levers (P3–P4) and the DUT lever (P6 or P8).

#### 5.3.4 Mount the tested servo and puller servos on the base plate

1. Position the tested-servo holder in the center of the base plate.
2. Attach the holder using clamps or appropriate fasteners, depending on the base-plate material.
3. Position the puller-servo holders on both sides of the tested servo assembly.
4. Adjust the distance so that the elastic bands run in straight lines and remain only lightly tensioned at neutral (vertical lever position).
5. Secure the puller-servo holders using clamps. You may also secure mounts using M4 hardware (F4–F19).

Verify that the lever is roughly vertical—this is the neutral (home) position.

#### 5.3.5 Elastic-band load calibration

The elastic bands should apply enough tensile force to reliably seat the gear train on opposite flanks during clockwise and counter-clockwise loading. In our setup, approximately 0.15 kgf in the pulled state was sufficient for the single-servo DUT configuration, while approximately 0.3 kgf was sufficient for the dual-servo DUT configuration. The force can be checked using a small hanging scales as described in Section 5.2.4 by measuring the band tension parallel to the ground and adjusting the lever-to-lever distance (band extension) accordingly. High absolute accuracy is not required; the primary requirement is that the load is sufficient and approximately symmetric in both directions. Since backlash is computed from encoder readings taken when both elastic bands return to the relaxed state, small variations in the exact force level do not significantly affect the measurement, provided the gear train is consistently preloaded during each pull.

**Fig. 5:**
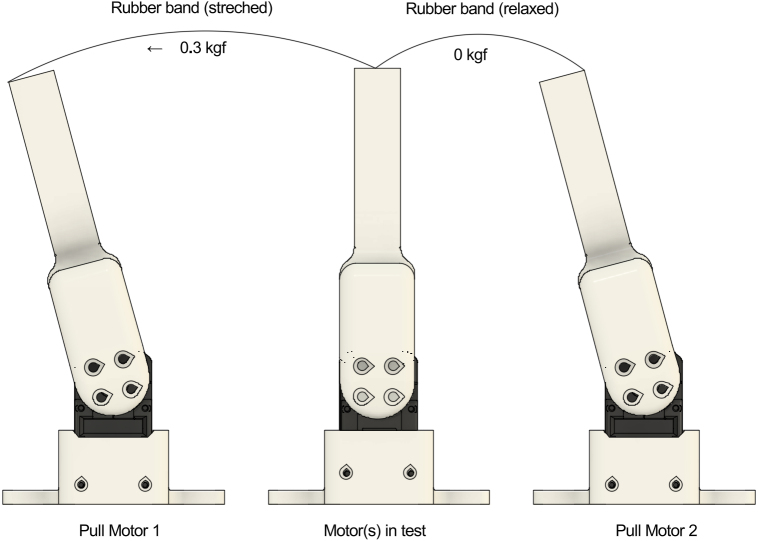
Example test stand setup (dual DUT) and two puller servos mounted on a base plate.

Fig. 5 shows an example configuration with two tested servos (dual DUT) and two puller servos.

#### 5.3.6 Connect the servos electrically

All servos — puller motors and the tested motor or motors — are connected in a daisy-chain manner using their UART ports. This creates a single shared communication bus with only three required lines: VCC (typically 7.4 or 12 V, depending on servo model), GND and UART signal (half-duplex, TTL-level)

1. Daisy-chain the servos using the supplied cables. Each servo has two identical UART ports. There is no In/Out distinction; connect any port to the next servo.
2. Connect one of the servos (e.g., a puller motor M1) to the USB–TTL adapter (U1).

**Fig. 6:**
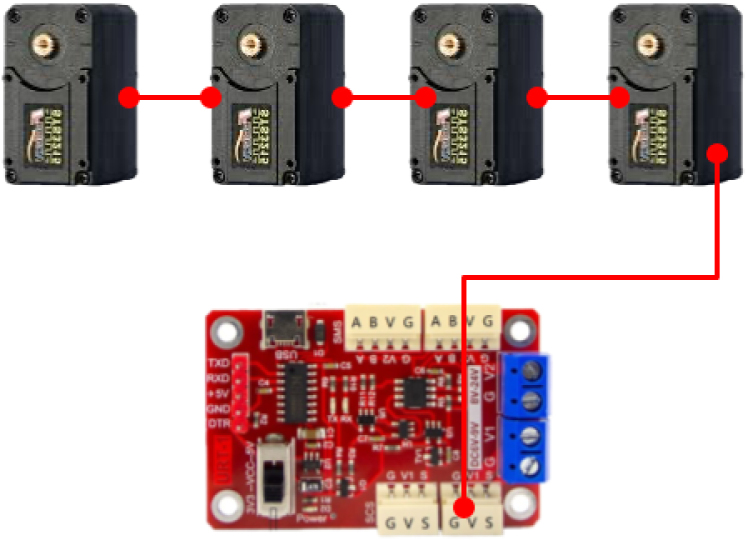
TTL half-duplex daisy-chain bus topology used in this work (single shared serial bus; common power distribution).

The wiring scheme used in this work is illustrated in Fig. 6.

#### 5.3.7 Power and computer connection

The stand can be powered by a standard DC supply appropriate for the selected servo models (commonly 12 V for Feetech or Waveshare units).

1. Connect the DC power supply (PS1) to the power input on the USB–TTL adapter/breakout (U1).
2. Connect the USB–TTL adapter (U1) to the PC using a USB cable.

A 2 A power supply is sufficient for this stand because the servos are not operated near stall torque and do not draw peak current during the test sequence. Typical current draw per servo is on the order of 100–200 mA, so the total steady-state current for four servos remains well below 2 A with reasonable margin.

### 5.4 Calibration

Calibration ensures that all servos start from a consistent neutral (middle) position before running any tests.

**Fig. 7:**
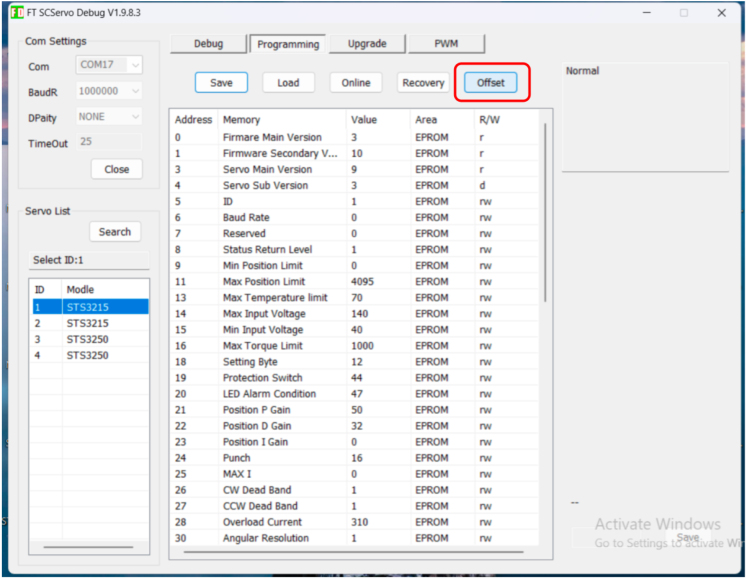
Feetech configuration utility.

1. Open the Feetech Utility (FD 1.9.8.3).
2. Select the appropriate COM port, set the baud rate (typically 1,000,000 bps), and click **Connect**.
3. Click **Scan** to detect all connected servos on the UART bus.
4. For each servo:
  • Manually rotate each servo’s lever so it is in the vertical (neutral) position.
  • Open the Programming tab and click **Offset** (see Fig. 7).

After calibration, the stand is ready for running the automated test sequence described in Section 6.

## 6 Operation instructions

### 6.1 Software overview

**Fig. 8:**
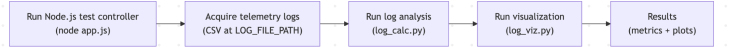
Software worlflow used in this work.

The software stack coordinates the full test workflow: it drives the UART servos, executes the motion sequence, records telemetry, and provides tools for analysis and visualization (see Fig. 8). The system is intentionally lightweight and runs on a standard PC (or single-board computer) through a simple USB–to–TTL connection. All components are open source and designed for easy modification.

#### 6.1.1 Test controller

The test controller is a Node.js application that communicates with all servos over the shared UART bus and executes the predefined motion sequence. Its main responsibilities include:

• **Test sequencing:** running the preload motion, applying calibrated tension through the puller servos, alternating between clockwise (CW) and counter-clockwise (CCW) pulls, and relaxing the load between phases.
• **Synchronized control:** ensuring all motors execute their portion of the sequence in a coordinated and repeatable manner.
• **Telemetry logging:** continuously collecting status data from each servo during the full test cycle.

During test sequence, each servo reports its state at regular intervals. The controller collects these packets and stores them in CSV format. Typical fields include: Timestamp, Servo ID, Target (commanded) position, Actual encoder position, Estimated load/torque, Motor current, Supply voltage.

The default logging rate is approximately 10 Hz but can be increased or decreased depending on test conditions. These data form the basis for quantifying backlash, tracking positional deviations, and evaluating servo behavior under varying loads.

#### 6.1.2 Analysis tools

A Python script is provided for processing and interpreting the recorded telemetry. Its core functions include:

• **Phase detection:** using puller-servo positions to identify preload, loaded, and unloaded intervals.
• **Position-deviation analysis:** comparing target and actual positions under different loading conditions.
• **Backlash estimation:** extracting CW and CCW reference points to compute effective backlash.
• **Averaging and statistics:** aggregating multiple cycles to obtain stable, repeatable measurements.

The output includes numerical summaries and intermediate datasets suitable for further study or comparison across servo models and parameter settings, as used in Section 7.6.

#### 6.1.3 Visualization tools

A dedicated visualization script converts processed data into publication-ready charts. It produces plots such as:

• Encoder position vs. time
• Target position vs. time
• Loaded and unloaded phases highlighted over the time series

These visualizations make it easier to interpret servo behavior, observe how different configurations influence performance, and communicate results effectively.

### 6.2 Software installation

The test controller is located in the repository under:

**Figure:**
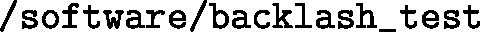

The controller is a Node.js application and requires Node.js and npm to be installed on the host computer.

1. Install Node.js (version 18 or later recommended).
2. Install npm (included with most Node.js distributions).
3. Open a terminal and navigate to the test controller directory:

**Figure:**
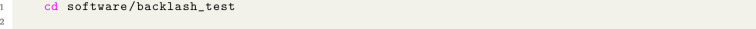
4. Install all required dependencies:

**Figure:**
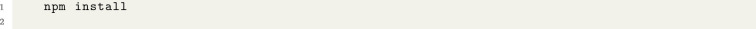

### 6.3 Configuration

Put hardware configuration, such as serial port address and baud rate in **.env** file:

**Figure:**
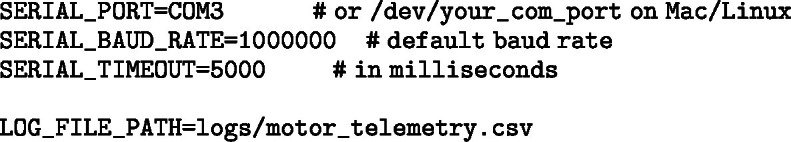

Test behavior is controlled through the configuration file:

sweepConfig.js

In this file, you can adjust:

• Servo IDs (tested servos and puller servos)
• Motion positions (neutral, preload, pull distances)
• Timing parameters (delays, cycle durations, settling times)
• Speed and acceleration for both tested motors and pull motors

These values must match your mechanical setup and desired test conditions. For example, ensure that:

• Motor IDs correspond to the IDs assigned during calibration (Section 5.4).
• Puller motors are given appropriate acceleration so that they tension elastic bands smoothly.
• Tested motors use safe speed limits to avoid overshoot.

### 6.4 Default motion sequence

The default configuration assumes:

Motors 1 and 2 — tested servo(s) Motors 3 and 4 — puller motors

**Fig. 9:**
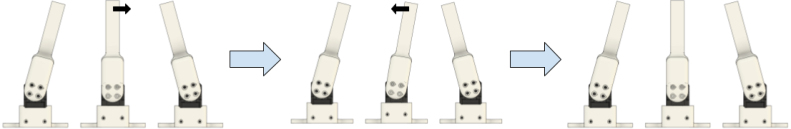
Test sequence, stage 1: preparation. Both pull elastic bands are relaxed. The tested motor(s) moves in CW or CCW direction and returns back in center position. This causes motor gears to preload.

**Fig. 10:**
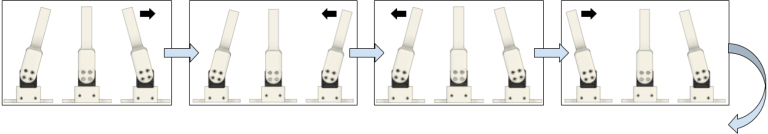
Test sequence, stage 2: stress. The tested motor(s) lever remains in the central position. The pull motors alternately pull the tested motor(s) lever in opposite directions. Between pulls, both elastic bands are relaxed.

The tested motor(s) executes a short back-and-forth movement to preload its internal gears either CW or CCW (Fig. 9). Once loaded, the puller motors alternately pull the lever in opposite directions. Between each pull, they briefly release tension to ensure that no residual force biases the measurement (Fig. 10).

### 6.5 Running the test controller

Once configuration is complete, the controller can be started with:

**Figure:**
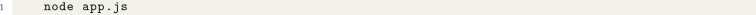

Log file will be saved automatically under

**Figure:**
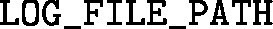

if configured in **.env** or default log path at

**Figure:**
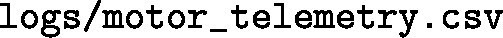

### 6.6 Python log-analysis tools

Additional analysis and visualization tools are provided in:

**Figure:**
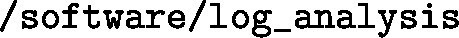

These scripts require standard Python scientific libraries (e.g., NumPy, Pandas, Matplotlib). Using a virtual environment is recommended but optional.

A minimal dependency installation can be done with:

**Figure:**
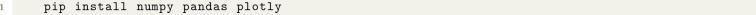

These scripts process the CSV logs produced by the controller, compute backlash-related metrics, and generate visualizations for further interpretation.

### 6.7 Log-analysis workflow

Two main Python tools are provided for analyzing backlash and visualizing results: **log_calc.py** and **log_viz.py**.

#### 6.7.1 log_calc.py — Backlash analysis

log_calc.py processes a log CSV together with a JSON configuration file. The configuration specifies:

• Which motors define the analysis-phase window (e.g., puller-motor positions)
• Which motors should appear in the output report
• Reference encoder values for home, loaded, and unloaded states
• Motor IDs for both tested and puller servos
• Expected relaxed/stretched positions for force-application events

Run the script as follows:

**Figure:**
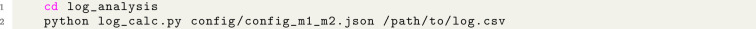

The script outputs numerical measurements such as effective backlash, loaded/unloaded offsets, and statistical summaries.

#### 6.7.2 log_viz.py — Visualization Tool

log_viz.py uses the same JSON configuration files. It reads the log and generates interactive Plotly-based visualizations.

It uses:

• report_motor_ids to decide which motors to plot
• Puller-motor activity to overlay relaxed and stretched intervals
• Reference positions to highlight important transitions

Run it the same way:

**Figure:**
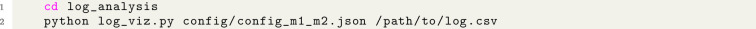

#### 6.7.3 Configuration files

All configuration files are stored in:

**Figure:**
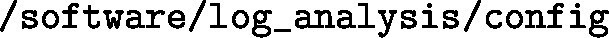

Each JSON file can define:

• Motor IDs (tested motors, puller motors)
• Reference positions for home, loaded, and unloaded states
• Analysis-window parameters
• Reporting options

These configs allow a single log-analysis workflow to support different test setups (single motor, dual motors, different loading strategies), as used in the measurements summarized in Section 7.

## 7 Validation and characterization

To validate the test stand and demonstrate its ability to quantify backlash behavior, we evaluated several UART servo configurations commonly used in our projects. The tested units included:

• STS3215 (single-servo configuration)
• STS3215 (dual-servo coupled configuration)
• STS3250 (single-servo configuration)

### 7.1 Backlash measurement procedure

As part of each test, a sequence of motions is executed as specified in Section 6.4. During this sequence, motor telemetry (including each servo current position and target position) is sampled at approximately 10 Hz rate.

During measurement, the tested servo(s) are commanded to hold a fixed target position to prevent backdriving. The elastic-band load is calibrated to seat the gear train in both CW and CCW directions without overcoming the holding torque (Section 5.3.5).

For each pull direction (CW and CCW), two reference points are extracted from the telemetry stream:

#### Unloaded (relaxed) state.

the actual encoder position immediately after both pull motors release tension.

#### Loaded (stretched) state.

the actual encoder position while the approximately 0.3 kgf force is applied in that direction.

For each of these states, multiple samples are collected over the repeated motion cycles. The samples are averaged to obtain stable estimates of:

• $p_{\mathrm{cw,unloaded}}$
• $p_{\mathrm{ccw,unloaded}}$
• $p_{\mathrm{cw,loaded}}$
• $p_{\mathrm{ccw,loaded}}$

The total positional deviation between the two pull directions is then computed separately for the unloaded and loaded conditions:

(1) $$d_{\mathrm{unloaded}}=|p_{\mathrm{cw,unloaded}}-p_{\mathrm{ccw,unloaded}}|$$

(2) $$d_{\mathrm{loaded}}=|p_{\mathrm{cw,loaded}}-p_{\mathrm{ccw,loaded}}|$$

These two values represent the measured backlash in the absence of external force and under the applied counter-torque, respectively.

Since the motion sequence is repeated multiple times, a sufficient number of samples is collected to compute the average backlash and its dispersion for each configuration (single motor, dual motor, dual motor with preload).

A 10 Hz sampling rate is sufficient because the analysis uses only steady-state portions of each phase (loaded and unloaded) and averages samples collected while the encoder position is sustained, rather than capturing transient transition dynamics.

### 7.2 Geometric and force relationships

For this stand, it is useful to relate three quantities:

• Angular motion at the servo shaft,
• Linear displacement at the lever tip,
• Torque applied by a known force at the lever tip.

#### 7.2.1 Lever tip displacement and angular backlash

For a lever of length r and a small angular change Δθ, the linear displacement Δs at the tip is approximately

(3) Δs≈rΔθ,

where r is in metres and Δθ is in radians.

Using degrees is often more practical. With

(4) $$\Delta \theta_{\text{rad}}=\Delta \theta_{\text{deg}}\cdot \frac{\pi }{180},$$

we obtain

(5) $$\Delta s=r\cdot \frac{\pi }{180}\;\Delta \theta_{\text{deg}}.$$

A typical UART hobby servo uses a 12-bit output encoder with a resolution of 4096 counts per revolution for the class of UART servos considered in this work [1], [2]. This corresponds to an angular step size of

(6) $$\Delta \theta_{\text{enc}}=\frac{360^{\circ }}{4096}\approx 0.088^{\circ }\text{per count}.$$

For the 100 mm lever used in our test stand (r=0.1m), this angular increment translates to a linear displacement at the lever tip of

(7) $$\Delta s_{\text{enc}}=r\cdot \frac{\pi }{180}\;\Delta \theta_{\text{enc}}\approx 0.153\;\text{mm per count}.$$

These values define the smallest position change that can be observed directly from the servo’s reported encoder position and therefore set a practical lower bound on the backlash that can be reliably resolved with this measurement method.

This provides a simple way to interpret encoder-based backlash (in degrees or encoder steps) as physical motion at the end of the lever, and forms the basis for translating the results in Section 7.

#### 7.2.2 Force at the lever tip and torque at the shaft

The torque τ at the servo shaft is the product of the force F at the lever tip and the lever arm length r:

(8) τ=F⋅r.

If the force is measured with a small scale in kilogram-force (kgf) and the lever length is expressed in centimetres, the torque in kg cm is

(9) $$\tau_{\text{kg cm}}\approx F_{\text{kgf}}\cdot r_{\text{cm}}.$$

As an example, for a 100 mm (10 cm) lever and a measured pull of 0.15 kg at the tip:

(10) $$\tau_{\text{kg cm}}\approx 0.15\times 10=1.5\;\text{kg cm}.$$

In SI units this is approximately 0.147  N m. This relationship makes it straightforward to calibrate different load levels by adjusting either the lever length, the band tension, or both.

### 7.3 Single STS3215 servo configuration

The motor was mounted rigidly, driven to a fixed target position (2047), and subjected to the standard force-application sequence described earlier.

Analysis script output:

**Figure:**
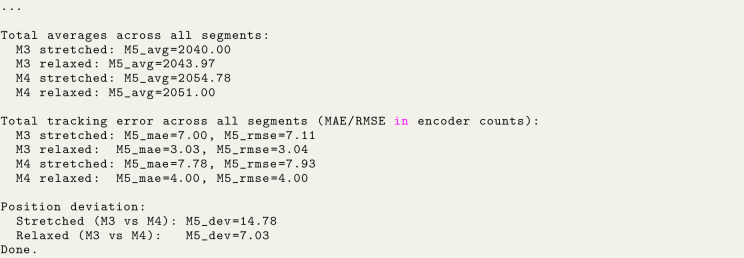

**Fig. 11:**
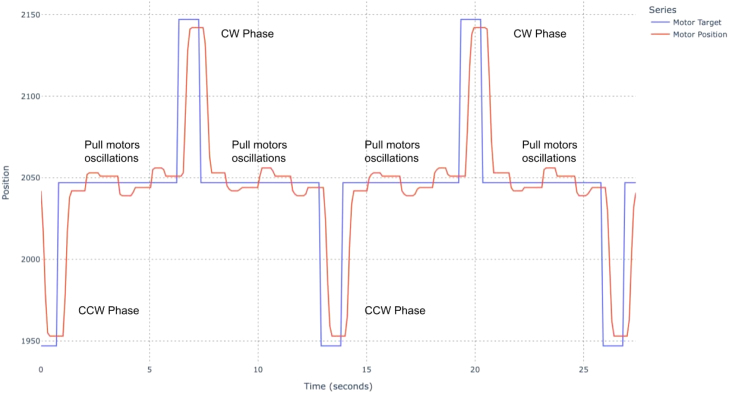
Test results of a single STS3215 with default settings (y-axis in encoder counts).

These correspond to (using the geometric relationships in Section 7.2.1):

• Loaded backlash: ≈1.30° or ≈2.27mm at 100 mm radius,
• Unloaded backlash: ≈0.62° or ≈1.08mm at 100 mm radius.

In addition, steady-state tracking error during the loaded and unloaded phases is quantified via MAE/RMSE between commanded and measured encoder position. For the single-servo configuration, the worst-case steady-state errors across pull directions are MAE ≈7.78 counts (loaded) and 4.00 counts (unloaded), with RMSE ≈7.93 counts (loaded) and 4.00 counts (unloaded).

These results confirm that a single STS3215 exhibits on the order of 1–2 mm of effective backlash at a 100 mm lever radius, increasing under load (see Fig. 11), which motivates the use of dual-servo configurations and backlash compensation strategies described in the following sections.

### 7.4 Two coupled STS3215 servos

To evaluate the effect of mechanically coupling two STS3215 servos, the motors were tested in a rigid back-to-back configuration without applying any positional offset or deliberate pretension. Both actuators were commanded to the same target position and driven synchronously, sharing a common output through the fixed bracket assembly.

Analysis script output:

**Figure:**
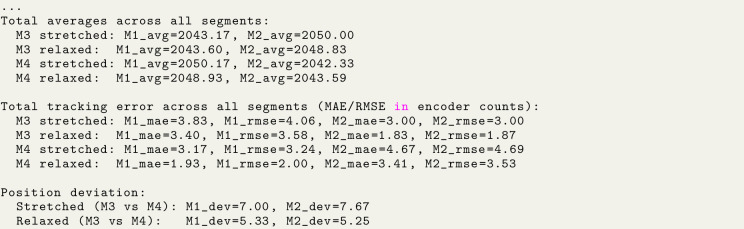

**Fig. 12:**
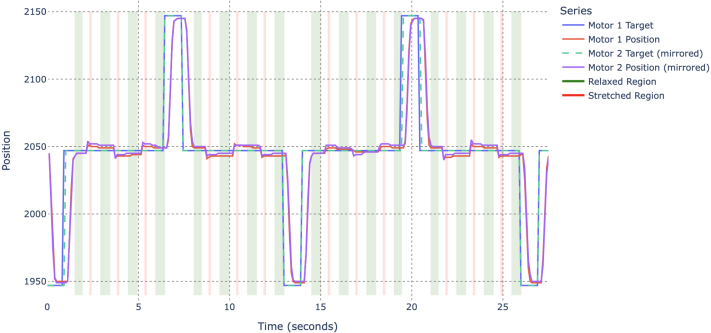
Test results of two coupled STS3215 motors with default settings. Pull-motor relaxed and stretched regions are outlined for reference.

With two STS3215 servos rigidly coupled and commanded to the same target position, the effective backlash is reduced compared to a single actuator. Using the relationships in Section 7.2.1, the CW–CCW deviation is:

#### Loaded (∼3kg cm).

• Servo 1: 7.00 counts $\approx 0.62^{\circ }\approx 1.07\;\text{mm}$ at 100 mm radius
• Servo 2: 7.67 counts $\approx 0.67^{\circ }\approx 1.17\;\text{mm}$ at 100 mm radius

#### Unloaded.

• Servo 1: 5.33 counts $\approx 0.47^{\circ }\approx 0.82\;\text{mm}$ at 100 mm radius
• Servo 2: 5.25 counts $\approx 0.46^{\circ }\approx 0.80\;\text{mm}$ at 100 mm radius

Steady-state tracking error is also reduced compared to the single-servo case (see Fig. 12). Reporting worst-case values across both servos and both pull directions, the coupled configuration yields MAE ≈4.67 counts (loaded) and 3.41 counts (unloaded), with RMSE ≈4.69 counts (loaded) and 3.58 counts unloaded).

### 7.5 Single STS3250 motor

**Fig. 13:**
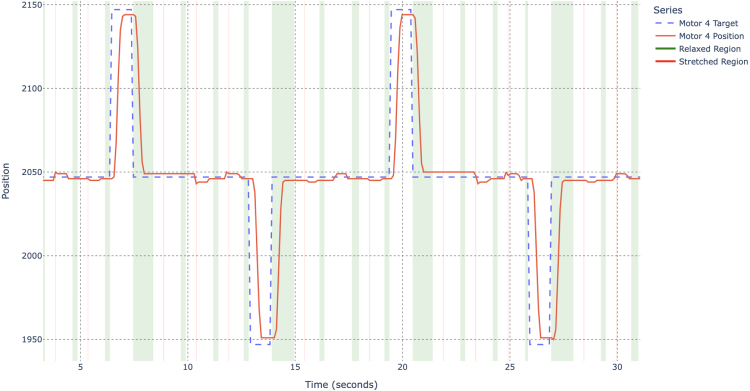
Test results of single STS3250 with default settings.

Finally, a single STS3250 motor was tested using the same procedure (see Fig. 13). The resulting loaded and unloaded deviations fell within the range expected from the manufacturer’s datasheet [3], confirming that the stand accurately captures the motor’s nominal backlash characteristics.

**Figure:**
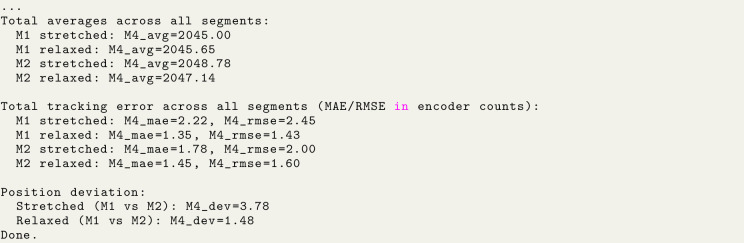

These correspond to (using the geometric relationships in Section 7.2.1):

• Loaded backlash: 3.78 counts ≈0.33° or ≈0.58mm at 100 mm radius,
• Unloaded backlash: 1.48 counts ≈0.13° or ≈0.23mm at 100 mm radius.

### 7.6 Results summary

To enable direct comparison among all test configurations, backlash values for both loaded (L) and unloaded (U) conditions were compiled into a single results table. For dual-motor setups, the larger (worst-case) deviation between the two servos is used to represent system backlash.

**Fig. 14:**
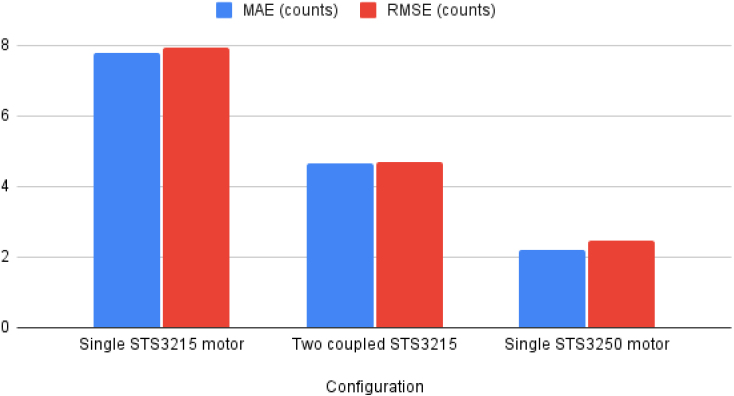
Steady-state tracking error over all detected loaded/unloaded segments, reported as mean absolute error (MAE) and root mean square error (RMSE) in encoder counts for three test configurations.

The consolidated results in Table 4 show that the proposed test stand can resolve differences in backlash across actuator types and configurations under repeatable bidirectional loading. Table 5 complements this by quantifying steady-state tracking error (MAE/RMSE), indicating that the measured CW/CCW offsets are not dominated by control noise. Together, these results support the objective of quantifying backlash and comparing configurations using robust, reproducible metrics. Fig. 14 visualizes the MAE and RMSE tracking error values reported in Table 5 for the three tested configurations.

**Table 4:** Combined backlash test results. U: unloaded (measured immediately after both pulling motors relax). L: loaded (measured while a counter-force is applied). Backlash is expressed in encoder counts, angular displacement, and linear displacement at a 100 mm radius. For two-motor setups, the larger of the two measured deviations is reported.

Configuration	Backlash (counts)	Backlash (deg)	Backlash (mm)
Single STS3215 motor (U)	7.03	0.62	1.08
Single STS3215 motor (L)	14.78	1.30	2.27
Two coupled STS3215 (U)	5.33	0.47	0.82
Two coupled STS3215 (L)	7.67	0.67	1.17
Single STS3250 motor (U)	1.48	0.13	0.23
Single STS3250 motor (L)	3.78	0.33	0.58

**Table 5:** Steady-state tracking error over all detected loaded/unloaded segments, reported as mean absolute error (MAE) and root mean square error (RMSE) in encoder counts. For two-motor setups, the worst-case motor value is reported.

Configuration	MAE (counts)	RMSE (counts)
Single STS3215 motor	7.78	7.93
Two coupled STS3215	4.67	4.69
Single STS3250 motor	2.22	2.45

The analysis results were instrumental in guiding our design decisions. By directly comparing backlash performance across servo types and configurations, we were able to determine which approach offered the most stable and predictable behavior for our intended application. Ultimately, the measured data supported selecting a specific servo configuration for use in our upcoming product.

## Human and animal rights

This work did not involve human subjects or animals.

## Declaration of Generative AI and AI-assisted technologies in the writing process

During the preparation of this work, the author used OpenAI ChatGPT in order to assist with text structuring, language improvement, and refinement of technical descriptions. After using this tool, the author reviewed, verified, and edited all generated content and takes full responsibility for the content of the published article.

## Funding

This research did not receive any specific grant from funding agencies in the public, commercial, or not-for-profit sectors.

## Declaration of competing interest

The authors declare the following financial interests/personal relationships which may be considered as potential competing interests: The authors are employees of Robonine (https://robonine.com). The work described in this article was conducted as part of their employment. Robonine may benefit from the dissemination of the results for educational and public outreach purposes.

## References

[1] Shenzhen Feetech RC Model Co., Ltd. (2023). https://www.feetechrc.com/Data/feetechrc/upload/file/20240507/6385067068652648096680943.pdf.

[2] Waveshare Electronics (2023). https://www.waveshare.com/w/upload/f/f4/ST3215_Servo_User_Manual.pdf.

[3] Shenzhen Feetech RC Model Co., Ltd. (2023). https://www.feetechrc.com/Data/feetechrc/upload/file/20240507/6385067068652648096680943.pdf.

[4] Mitutoyo Corporation (2024). https://www.mscdirect.com/product/details/62498175.

[5] Mitutoyo Corporation (2024). https://www.higherprecision.com/products/indicators/mitutoyo-3415a-series-3-large-dial-face-dial-indicator-0-_500-inch-range-lug-back-1_8n-or-less-force.

[6] Stein J.L., Wang C.-H. (1998). Estimation of gear backlash: Theory and simulation. J. Dyn. Syst. Meas. Control..

[7] Chandrasekar P., Srinivasan K. (2021). Inferential based measurement of backlash in servo system. Mater. Today: Proc..

[8] Yang Q., Liu Y., Zhu Z. (2021). A planetary gear reducer backlash identification based on servo motor current signal and optimized fisher discriminant analysis. ISA Trans..

[9] Gleason Corporation (2024). https://www.gleason.com/en/products/metrology/metrology-systems/analytical-inspection-and-cmm/475gms-to-3000gms-inspection-solutions-for-medium-sized-and-larger-gears.

[10] Kudale Instruments Pvt. Ltd. (2024). http://www.kudaleinstruments.com/calibration-laboratory/dimentional-metrology.html.

[11] Shenzhen Feetech RC Model Co., Ltd. (2025). https://gitee.com/ftservo.

